# Impact of a total lockdown for pandemic SARS-CoV-2 (Covid-19) on deep surgical site infections and other complications after orthopedic surgery: a retrospective analysis

**DOI:** 10.1186/s13756-021-00982-z

**Published:** 2021-07-31

**Authors:** Ines Unterfrauner, Laura A. Hruby, Peter Jans, Ludwig Steinwender, Mazda Farshad, Ilker Uçkay

**Affiliations:** 1 Department of Orthopedics, Balgrist University Hospital, University of Zurich, 8008 Zurich, Switzerland; 2 Medical Informatics Service, Balgrist University Hospital, University of Zurich, 8008 Zurich, Switzerland; 3 Infection Control, Balgrist University Hospital, University of Zurich, 8008 Zurich, Switzerland; 4 Unit of Clinical and Applied Research, Balgrist University Hospital, University of Zurich, 8008 Zurich, Switzerland; 5 Balgrist University Hospital, University of Zurich, Forchstrasse 340, 8008 Zurich, Switzerland

**Keywords:** SARS-CoV-2, Covid-19, Total lockdown, First epidemic wave, Pandemic, Orthopedic surgery, Deep surgical site infections, Wound healing disorders, Postoperative complications, Healthcare-associated infections

## Abstract

**Background:**

A total lockdown for pandemic SARS-CoV-2 (Covid-19) entailed a restriction of elective orthopedic surgeries in Switzerland.  While access to the hospital and human contacts were limited, hygiene measures were intensified. The objective was to investigate the impact of those strict public health guidelines on the rate of intra-hospital, deep surgical site infections (SSI), wound healing disorders and non-infectious postoperative complications after orthopedic surgery during the first Covid-19 lockdown.

**Methods:**

In a single-center study, patients with orthopedic surgery during the first Covid-19 lockdown from March 16, 2020 to April 26, 2020 were compared to cohorts that underwent orthopedic intervention in the pre- and post-lockdown periods of six months each. Besides the implementation of substantial public health measures (promotion of respiratory etiquette and hand hygiene), no additional infection control bundles have been implemented.

**Results:**

5791 patients were included in this study. In multivariate Cox regression analyses adjusting for the large case-mix, the lockdown was unrelated to SSI (hazard ratio (HR) 1.6; 95% confidence interval (CI) 0.6–4.8), wound healing disorders (HR 0.7; 95% CI 0.1–5.7) or other non-infectious postoperative complications (HR 0.7, 95% CI 0.3–1.5) after a median follow-up of seven months.

**Conclusion:**

The risks for SSI, wound healing disorders and other complications in orthopedic surgery were not influenced by the extended public health measures of the total Covid-19 lockdown.

*Trial registration* BASEC 2020–02646 (Cantonal Ethics Commission Zurich).

*Level of evidence*: Level III.

## Background

On March 11, 2020, the World Health Organization declared a worldwide pandemic due to the extremely rapid expansion of SARS-CoV-2 (Covid-19) infections. Besides its highly contagious profile, severe consequences due to acute respiratory syndrome and unpredictable sequelae on the overall health status, the socio-economic impact and financial burden on healthcare systems have been significant and will keep challenging societies all over the world [1, 2]. Many health authorities introduced a nationwide, total lockdown together with a bundle of various hygienic measures [1, 2]. In Switzerland, the first total lockdown implemented a panoply of public health measures between March 16, 2020 and April 26, 2020. All elective surgeries were restricted to spare medical professionals and secure resources for severely ill patients. The number of surgical procedures and the number of hospitalizations decreased significantly [3]. On the other hand, the Covid-19 pandemic led to intensified hygiene awareness and measures with increased hand-rubbing and constant use of surgical masks and gloves. With an increased awareness of viral infections in the community and hospitals, we hypothesized that those public health bundles yielded a positive impact on classical postoperative complications such as deep surgical site infections (SSI), other healthcare-associated infections (HAI), postoperative complications, and on the observed hand hygiene (HH) compliance [4].

To the best of our knowledge, no study to date has investigated the impact of the Covid-19 lockdown on postoperative infections, wound- and other surgery-related complications in orthopedic surgery. Hence, we aimed to fill this gap with a single-center cohort and aspired to elucidate the impact of enhanced hygiene measures and limited human contacts on many important complications after orthopedic surgery. We renounced on reporting HAI among hospitalized Covid-19 patients and avoided to analyze the nosocomial proportion of Covid-19, for which a broader literature is available.

## Methods

### Setting

The Balgrist University Hospital prospectively registers all moderate to severe infections, including many postoperative complications, since July 2018. The immediate pre-lockdown period witnessed no specific Covid-19 policies besides the promotion of the "respiratory etiquette" and HH [5–9]. During the lockdown, the authorities banned all elective surgeries and visitors in hospitals. They implemented social distancing and home office for healthcare workers (HCW) at risk [2]. Hence, during the lockdown, only emergency patients were treated, or patients with multiple co-morbidities transferred from other hospitals to release capacities for their severely ill Covid-19 patients. Importantly, a mandatory mask use was introduced only after the first epidemic wave, as was Contact Tracing and the post-exposition quarantine for asymptomatic HCW with close contact to Covid-19 positive persons.

### Data collection and study criteria

**Fig. 1:**
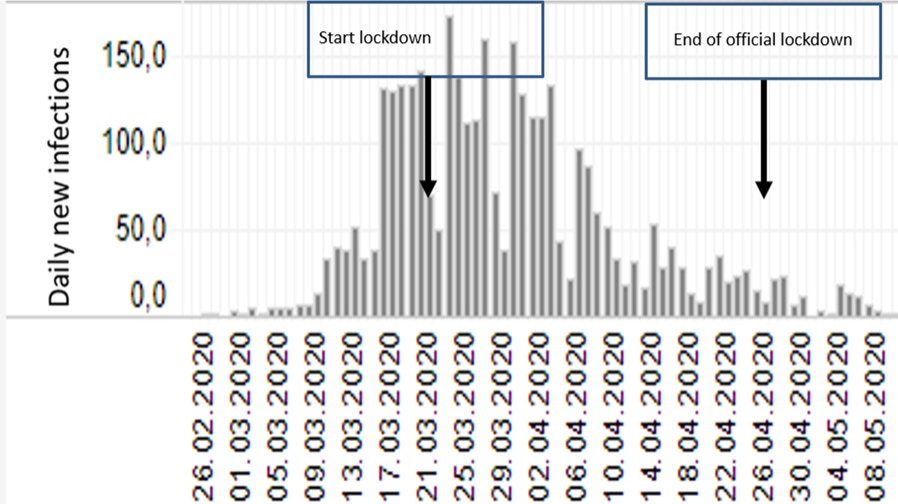
The epidemic curve of the first wave of Covid-19 in Switzerland. Adapted from reference [3]

This study followed the ethical principles of the Helsinki Declaration and approval of the project was obtained from the Cantonal Ethics Commission. A one-year study period (October 1, 2019–October 31, 2020) was arbitrarily chosen, was divided into three periods and included all surgeries performed in the operation theater: pre-lockdown period with 2688 interventions from October 1, 2019 to March 15, 2020; the Covid-19 lockdown period with 230 surgeries from March 16, 2020 to April 26, 2020 (Fig. 1); and a post-lockdown period with 2873 interventions from April 27, 2020 to October 31, 2020. The most important reason regarding the choice for this precise study period was the stability of the surgical teams, lasting on average one academic year. Similarly, the one-year study period, six months before and six months after the lockdown, guaranteed the continuity of the operating personnel. Additionally, for this chosen period, the accuracy of the perioperative prophylactic antibiotic regimens could be verified. Finally, the two control periods (before and after lockdown) reduced the selection bias compared to only one control period (Fig. 1).

All adult patients undergoing orthopedic surgery at our institution were included. A general consent form allowed the registration of healthcare data for scientific usage signed by all included patients. The following exclusion criteria were used: adolescent patients, patients without agreeing to the general consent, and surgeries with a minimal active follow-up of less than 30 days. Database closure was on November 30, 2020. On this time, Switzerland already entered the second pandemic wave.

### Literature search

A literature review was performed to compare our orthopedic results of deep SSI, HAI and HH performances during the first lockdown for pandemic Covid-19 with available publications from other surgical and non-surgical specialties. For the literature search in English language, the following MeSH terms in PubMed and on the internet were used: "nosocomial infection", "healthcare associated infection", "surgical site infections", "lockdown", "hand hygiene", and "Covid".

### Outcome parameters, setting and definitions

The primary outcome was the incidence of deep SSI [10–12] after the index surgery. According to internationally accepted norms [10–12], SSI is defined as an event acquired in the operating theater manifesting within 30 days after the intervention, with drainage of purulent fluid out of the incision or presence of typical infectious signs (*rubor, calor, tumor, dolor*), and requiring revision surgery.

Secondary outcomes were the incidence of non-infectious postoperative wound healing disorders, postoperative local complications other than infection or visible wound problems, and the epidemiology of other non-surgical HAI between the three study periods. Wound healing disorders were defined as substantial necrosis, uninfected dehiscence without typical infectious signs and/or hematoma necessitating surgical drainage. Other causes for revision surgery were recurrence of disease, residual symptoms and/or intervention-specific complications. Key HAI were grouped as urinary tract infections, bacterial pneumonia, Covid-19 disease, and bloodstream infections [4]. Nosocomial infections attributed to other clinics were excluded. Additionally, the following parameters known to be associated with SSI [11] were documented: sex, age, body mass index (BMI), American Society of Anesthesiology (ASA)-Score, diabetes, date, types (including primary or revision surgery) and localization of index surgery, duration of surgery and length of hospital stay.

On the hospital's level, three collaborators with experience in infection control observed the HH compliance [5] at the beginning (October 2019) and the end of the study period (September 2020). There were no HH observations during the total lockdown. The HH observations were performed at the end of the study period to judge an eventual residual effect five months after the end of the lockdown. Additionally, the accuracy of the protocolled perioperative antibiotic prophylaxis was assessed. According to the intra-hospital standards, perioperative antibiotic prophylaxis was implemented with cefuroxime or with clindamycin (or vancomycin) in case of intolerance. The preoperative surgical skin site disinfection was performed with chlorhexidine or povidone-iodine. All operated patients were seen for a regular postoperative control 4–6 weeks after the index surgery.

### Statistical analysis

Group comparisons were performed using the Pearson-χ^2^ (categorical variables), the Wilcoxon-rank sum-test or the Kruskal–Wallis-test for non-parametric, continuous variables. To adjust for the heterogeneity of the surgeries and the large case-mix imposed by the ban of elective surgeries, three multivariate Cox regression analyses were performed. The three final Cox regression models targeted three different outcomes: "SSI", "wound healing disorders" and "other complications". In the first multivariate model, the group of SSI was compared to all other non-infected surgery episodes. In the second model, non-infectious wound problems were compared to all episodes without infection or wound problems, and in the last analysis, all local complications were compared to all uneventful surgeries. Most variables were analyzed as a continuum, but stratifications were added for the ASA-Score, the duration of surgery and the study period. The cut-off values for these strata relied on the 25%, 50% and 75% percentiles of the distribution of values of that variable. To adjust for the case-mix of the surgeries within the three study periods, we recurred to a multivariate Cox regression analysis by inserting stepwise independent variables with a *p* value ≤ 0.05 from the univariate analysis into the final model. The software IBM SPSS Statistics 25 and STATA™ (15.0, College Station, USA) were used and *p* values ≤ 0.05 (two-tailed) were considered significant.

## Results

### Study population

Within the one-year study period, 5791 orthopedic interventions were performed with a median follow-up of 218 days (SD 118) after the index surgery. The mean age of the overall study cohort was 55 years (SD 18; range 18–93) and 51% were males. 521 patients (9%) had diabetes mellitus, the mean BMI was 26.3 kg/m^2^ (SD 5.3) and the mean ASA-Score 2 points. The foot and ankle was the predominant surgery site (1118 interventions; 19%), followed by the knee (1103; 19%), the spine (1064; 18%), the shoulder (826; 14%), the hip (688; 12%), the hand (697; 12%), tumor surgery (132; 2%), and the diabetic foot (163; 3%). Of all index interventions, 47% were primary surgeries, the operative interventions lasted on average 85 minutes (SD 4) and the hospital stay averaged 3 days (SD 5). Considering re-interventions, 43 patients (0.7%) required revision surgery due to SSI, 39 patients (0.7%) due to wound healing disorders and 225 patients (3.9%) due to other causes. The mean time interval from index to revision surgery was 54 days and the average number of re-interventions was 1.2 surgeries.

### Comparisons between the study periods

According to exclusion criteria, 311 adolescent patients, 101 patients without agreeing to the general consent, and three patients with a minimal active follow-up of less than 30 days had to be excluded whereas all remaining patients were included for analysis. Except for the higher proportion of diabetic patients during the lockdown (12% vs. 8% vs. 10%; *p* = 0.02), the three time periods were comparable for demographics, BMI, ASA-Score, primary surgeries, the duration of surgery as well as the length of hospital stay (Table 1). Upon political order by health authorities, elective orthopedic interventions of patients with ASA-Scores exceeding 2 points had to be postponed during the first Covid-19 lockdown. Restrictions were eased again after the lockdown [2], accounting for the higher percentage of patients with an ASA-Score of 4 points in the post-lockdown period. The proportion of spine surgery was significantly higher during the lockdown (24% vs. 18%; *p* < 0.01) because of emergency operations performed in the Spine Center. Additionally, in crude group comparisons, the incidence of revisions for SSI was also significantly higher during lockdown (2% vs. 1% vs. 0.5%; *p* = 0.02) because of the emergency nature of surgical interventions. Non-infectious complications appeared significantly more often in the pre-lockdown period (5% vs. 3%; *p* < 0.01) whereas wound healing disorders were equally distributed between all three periods (Table 1).

**Table 1:** Crude group comparison of orthopedic surgeries before, during and after the lockdown

	Pre-lockdown	Lockdown	Post-lockdown	*p* values^a^
n = 5791	n = 2688	n = 230	n = 2873	
*Patients*				
Female sex	1311 (49%)	106 (46%)	1396 (49%)	0.74
Median age	53 years (18)	54 years (17)	53 years (18)	0.51
Diabetes mellitus	213 (8%)	27 (12%)	281 (10%)	***0.02***
Median body mass index	27.0 kg/m^2^ (5.3)	26.8 kg/m^2^ (6.0)	27.0 kg/m^2^ (5.3)	0.36
ASA-Score of 4 points	25 (1%)	5 (2%)	83 (3%)	***0.01***
*Types of orthopedic surgery*				***0.01***
Knee	526 (20%)	37 (16%)	541 (19%)	
Foot and Ankle	523 (20%)	24 (10%)	571 (20%)	
Spine	490 (18%)	56 (24%)	523 (18%)	
Shoulder and Elbow	385 (14%)	39 (17%)	402 (14%)	
Primary surgery	1250 (47%)	108 (47%)	1352 (47%)	0.66
Median duration of surgery	86 min (52)	89 min (53)	85 min (53)	0.33
Median duration of hospital stay	4.2 days (5.4)	4.5 days (7.3)	4.3 days (6.1)	0.17
*Postoperative outcomes*				
Revision for deep surgical site infection	26 (1%)	4 (2%)	13 (0.5%)	***0.02***
Revision for wound healing disorders	19 (0.7%)	1 (0.4%)	19 (0.7%)	0.88
Revision for non-infectious complication	138 (5%)	8 (3%)	79 (3%)	***0.01***
*Key non-SSI nosocomial infections*				
Bacterial pneumonia	9 (0.3%)	1 (0.4%)	13 (0.5%)	0.78
Urinary tract infections	7 (0.3%)	7 (3%)	13 (0.5%)	***0.01***
Secondary bloodstream infections	3 (0.1%)	1 (0.4%)	4 (0.1%)	0.44

Percentages respectively standard deviations are given in brackets

Significant results (*p* < 0.05) are indicated ***in bold and italic***

^a^Kruskal–Wallis-tests

### Other healthcare-associated infections and hand hygiene compliance

**Fig. 2:**
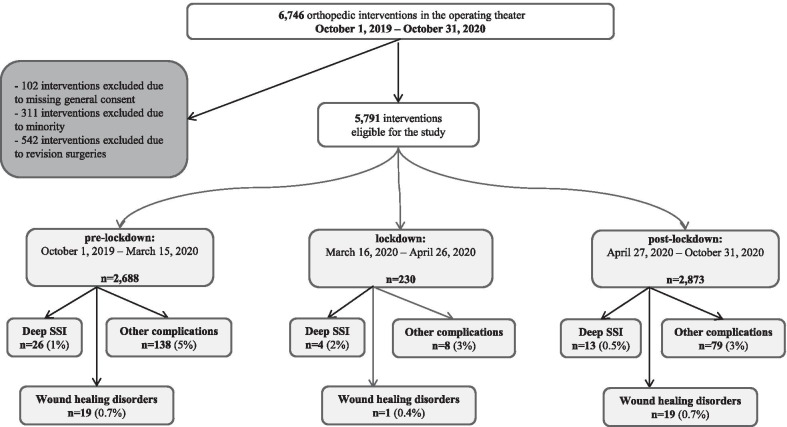
Flowchart of the analyses. SSI = surgical site infection; wound healing disorders = substantial necrosis, uninfected dehiscence and/or hematoma necessitating surgical drainage; other complications = recurrence of disease, residual symptoms and/or intervention-specific complications

The overall incidence of other HAI was 1.7% (99 infections per 5791 surgeries), including cutaneous or oral mycosis, urinary tract infections [13] and (aspiration) pneumonia [14]. None of our study patients was primarily hospitalized because of Covid-19, whereas the number of orthopedic patients with concomitant, symptomatic Covid-19 disease was 37 (0.6% of all episodes). Figure 2 reveals the study flowchart and the number of the various outcomes. The general compliance with the international HH recommendation was 75% in October 2019 (519 independent observations), and 70% in September 2020 (394 observations) [5]. This small difference was insignificant. We assessed the accuracy of the perioperative antibiotic prophylaxis at our institution for another concomitant study [15] by retrospectively controlling all SSI in the medical files. According to this analysis, a correct antibiotic prophylaxis including its timing occurred in 98% in all three study periods [15]. The HCW' staffing level was more than adequate (*data not shown*).

### Multivariate adjustment

Three individual Cox regression analyses assessed the influence of the lockdown period regarding the outcomes SSI (Table 2, left column), wound healing disorders (Table 2, middle column) and other non-infectious complications (Table 2, right column). In multivariate analyses, a duration of surgery > 0.8 h was associated with SSI (hazard ratio (HR) 3.6, 95% confidence interval (CI) 1.6–8.0) and a duration of surgery > 0.5 h with other complications (HR 1.7, 95% CI 1.2–2.5) (Table 2). For wound healing disorders, associations with the BMI (HR 1.1, 95% CI 1.0–1.1) and the operation duration > 0.5 h (HR 5.5, 95% CI 1.8–16.6) were shown. Undergoing surgery in the lockdown period lacked statistical interaction with all three outcomes SSI, wound healing disorders and other complications. Patients in the post-lockdown cohort had lower risks for both SSI (HR 0.4, 95% CI 0.2–0.8) and other complications (HR 0.5, 95% CI 0.3–0.6) but not for wound healing disorders.

**Table 2:** Univariate and multivariate associations with the stratified outcomes “SSI”, "non-infectious wound healing disorders" and "other non-infectious complications" (Cox regression analyses; results expressed as hazard ratios with 95% confidence intervals)

Variables	SSI (n = 43)		Wound healing disorders (n = 39)		Other complications (n = 225)	
	Univariate	Multivariate	Univariate	Multivariate	Univariate	Multivariate
Female sex	1.0, 0.6–1.8	1.1, 0.6–2.0	0.7, 0.3–1.3	0.6, 0.3–1.3	1.0, 0.8–1.3	1.0, 0.8–1.4
Age (*continuous*)	1.0, 1.0–1.0	1.0, 1.0–1.0	1.0, 1.0–1.0	1.0, 1.0–1.0	1.0, 1.0–1.0	1.0, 1.0–1.0
Diabetes mellitus	***2.7, 1.3–5.7***	2.1, 0.9–4.9	***2.6, 1.2–5.8***	1.4, 0.5–3.7	1.2, 0.7–1.8	0.8, 0.5–1.3
Body mass index (*continuous*)	1.0, 1.0–1.1	1.0, 0.9–1.1	***1.1, 1.1–1.2***	***1.1, 1.0–1.1***	***1.0, 1.0–1.1***	1.0, 1.0–1.0
ASA (*continuous*)	***1.7, 1.1–2.6***	-	***2.2, 1.4–3.4***	-	***1.4, 1.2–1.7***	-
ASA 2 compared to Score 1	1.4, 0.5–3.8	1.4, 0.5–3.9	3.0, 0.7–13.0	2.1, 0.4–9.9	1.1, 0.8–1.7	1.1, 0.7–1.7
ASA 3 compared to Score 1	***2.7, 1.0–7.5***	2.4, 0.7–8.5	***8.2, 1.9–35.1***	3.3, 0.6–19.0	***2.0, 1.3–3.0***	1.7, 1.0–3.0
ASA 4 compared to Score 1	3.9, 0.8–20.6	3.9, 0.6–26.9	4.9, 0.4–54.5	3.0, 0.2–44.1	1.8, 0.7–4.3	1.8, 0.6–5.2
Revision surgery	0.3, 0.1–2.0	-	0.5, 0.2–1.8	0.6, 0.2–2.1	1.0, 0.6–1.4	0.7, 0.3–1.5
Duration of surgery	1	-	1	-	1	-
0.5–0.8 h compared to < 0.5 h	1.9,0.8–4.3	1.9, 0.8–4.6	***5.1, 2.0–12.6***	***5.5, 1.8–16.6***	***2.0, 1.4–2.8***	***1.7, 1.2–2.5***
> 0.8 h compared to < 0.5 h	***3.8, 1.8–8.2***	***3.6, 1.6–8.0***	***3.8, 1.4–10.1***	***3.5, 1.1–11.5***	***2.9, 2.0–4.0***	***2.3, 1.6–3.3***
Study period	1	-	1	-	1	-
Lockdown vs. Pre-Lockdown	1.8, 0.6–5.2	1.6, 0.6–4.8	0.6, 0.1–4.6	0.7, 0.1–5.7	0.7, 0.3–1.4	0.7, 0.3–1.5
Post-lockdown vs. Pre-Lockdown	***0.5, 0.2–0.9***	***0.4, 0.2–0.8***	0.9, 0.5–1.8	0.9, 0.4–1.9	***0.5, 0.4–0.7***	***0.5, 0.3–0.6***

Statistically significant results are displayed ***in bold and italic***

In the first multivariate model, the group of SSI was compared to all other non-infected surgery episodes. In the second analysis, non-infectious wound problems were compared to all episodes without SSI or wound problems, and in the last analysis, all local complications versus all uneventful surgeries

“–“ = not included. SSI = surgical site infection

Of note, the goodness-of-fit tests were insignificant and the Receiver-Operating-Curve (ROC) value 0.72–0.76, reflecting an acceptable accuracy of the multivariate models. Table 3 resumes the key results of the literature search at one glance.

**Table 3:** Literature review of deep surgical site infections (SSI), other healthcare-associated infections (HAI) and hand hygiene performances during the first lockdown for pandemic Covid-19 (*limited to publications with own observed data*)

Journal	First author	Before lockdown	During lockdown	Remarks
*Reduced SSI or HAI*				
Asian Annals	Hussain et al. [15]	2.9%	0.8%	Sternal wound SSI after cardiac surgery
Updates Surg	Losurdo et al. [14]	3.4%	0.8%	General surgery in Trieste, Italy
J Neurol Sci	Cerulli Irelli et al. [26]	31.5%	23.3%	All HAI together in Stroke Units in Italy
Am J Infect Control	Wee et al. [17]	Baseline 100%	24%	Reduced catheter-related bacteremias
Am J Infect Control	Bentivegna et al. [28]	Baseline 100%	50–71%	Reduction of *Clostridium difficile*
Infect Control Hospital Epid	Ponce-Alonso et al. [27]	Baseline 100%	30%	Reduction of *Clostridium difficile*
*Stable SSI or HAI*				
Int J Infect Dis	Lo et al. [16]	Baseline	Baseline	All HAI together. Not quantified
J Orthop Surg Res	Zeng et al. [18]	1.0%	1.0%	SSI orthopaedic surgery in Shenzen, China
*Increased HAI*				
Am J Infect Control	McMullen et al. [19]	Baseline 100%	157–279%	Increase in urinary tract infections
Am J Infect Control	McMullen et al. [19]	Baseline 100%	327–420%	Catheter-related bloodstream infections
*Hand hygiene compliances*				
Am J Infect Control	Moore et al. [25]	46% compliance	56% compliance	No data on HAI or SSI; 19 hospitals in USA
Clin Microbiol Infect	Israel et al. [6]	46% compliance	80% compliance	No data on HAI or SSI; Covid-Units in Jerusalem
J Primary Care Comm Health	Roshan et al. [7]	unreported baseline	80–95%	No data on HAI or SSI. Mention of their reduction
Am J Infect Control	Wee et al. [17]	85%	100%	Reduction of selected HAI and of MRSA transmission

## Discussion

This large-scale study including 5791 adult orthopedic surgeries over one year in a tertiary university hospital was the first one to investigate the impact of Covid-19 lockdown on deep SSI, wound healing disorders and non-infectious complications following orthopedic surgery. The six-weeks total lockdown did not influence the risks of SSI, wound healing disorders, HAI or other postoperative complications after orthopedic interventions. We continued to witness HAI and observed a continuum of the HH compliance rather than an increased compliance several months after the lockdown [5–9].

The impact of strict lockdown measures has not been investigated in orthopedic surgery yet. Generally, the literature is sparse and the opinions divided considering the influence of a lockdown including intensified hygiene awareness, increased hand-rubbing and reduced human contacts on SSI and HAI. Earlier publications advocated a favorable outcome on SSI. To cite examples, a general surgery cohort compared 123 patients operated in the Covid-19 lockdown to 400 patients operated in the same time period one and two years earlier. The SSI risk was significantly lower during the lockdown [16]. Similarly, a study in the field of cardiac surgery showed significantly lower rates of sternal wound infections in 493 patients operated during lockdown compared to patients with surgical intervention in the twelve months preceding the lockdown [17]. All authors attributed this to intensified precautions and additional SSI preventing measures including wear of protective equipment and surgical masks, reduction of present staff during surgery, shortened hospital stay, decontamination and isolation between patients, and restriction of visitors at the hospital. Among other HAI, it seems that especially catheter-related bloodstream infections (CLABSI) were particularly prone to reduction by increased awareness to infections [18, 19].

Similar to our findings, other research groups found the same SSI and overall HAI risk before and during the lockdown periods [20] or even a possible increase of urinary tract or catheter infections [16]. However, this analysis included much lower patient numbers, considered the time period of Covid-19 spread and not of lockdown guidelines and did not contain a multivariate analysis to adjust for influencing variables. Many infection control teams reported to spend the majority of time to the efforts linked to the Covid-19 pandemic without having enough time to continue other prevention bundles [21, 22]. Similarly, in Central Europe, all federal HAI surveillance systems were interrupted during the pandemic wave.

Although intensified hand hygiene is known to reduce SSI rates [23], there was no decrease of SSI in our orthopedic cohort. This might be explained by some reasons. First, the overall SSI rate in general surgery oscillates at 8% [16], whereas orthopedic surgery is associated with an infection rate of only 3% [4] and shown to be even less at our institution with an overall rate of 0.8% (*Unterfrauner *et al.,* unpublished data*). In cardiac surgery, the wound infection rate in daily surgical routine is higher compared to our institution (3% vs. 0.7%). Since infections after orthopedic surgery (i.e. osteomyelitis, septic arthritis) are known to ensue dramatic secondary complications and inflict long-term deficits on function and general health [24], well-trained orthopedic surgeons may have been more aware of the strict hygiene requirements to prevent SSI in the perioperative setting even before the lockdown period. Hence, new rigorous hygiene guidelines in the perioperative surgery setting could be more effective in reducing wound infections in other surgical fields compared to orthopedic surgery, where those measures may have been more effective without implementation of stricter hygiene measures. Furthermore, most patients undergoing cardiac surgery belonged to the Covid-19 high-risk group and thereby followed stricter precautions themselves.

Additionally, there is no strong rationale that public health measures reduce intra-hospital HAI without a concomitant specific prevention program [11]. Alternatively, simple orthopedic interventions might be less prone to postoperative complications, whereas centers operating more severe cases might yield a higher risk of complications, including SSI and HAI. Of note, our center received multiple transfers of healthcare-intensive patients with more complex orthopedic problems compared to standard orthopedic patients [2]. Further, surgical procedures were reduced to emergency interventions, which are known to have a higher risk of developing SSI [25, 26].

It would be of great interest to link an observed HH compliance to the incidence of HAI during the pandemic wave [7, 8, 18–20]. In the Covid-19 era, unfortunately, many publications supporting HH do not provide own HAI data, and vice versa. The only exception is a research group from Singapore [19]. The authors increased the overall HH compliance from 85 to 100% during the few weeks of the lockdown with a consecutive fall of the incidence-density of CLABSI to 24% [19]. Most hospitals simply lacked time to monitor HH during the pandemic wave. In Central Europe, the authorities targeted the public safety policies on masks and social distancing, whereas the promotion of HH in the community and healthcare setting was mostly left to hospitals and professional associations.

Strengths of our study include the high number of 5791 surgeries, a prospective register of infections, two control cohorts pre- and post-lockdown and adjusted analyses. Besides its retrospective design, it has some limitations:

1. The study population only concerned adult orthopedic patients and SSI. We might have missed superficial infections, or wound disorders treated in the ambulatory setting without the need for revision surgery or surgical consultation.
2. We lack a continuous monitoring of the HH compliance during the lockdown. It is clear that during the initial phase of the lockdown, the HH compliance consequently improved [7, 27]. The most important achievement would be the sustained effect [6], which we could not obtain. Likewise, we renounced on surrogates of direct HH observations such as the consumption of hand disinfection [8, 18], self-evaluations per questionnaires or automated assessments [27].
3. Due to the relatively short period of the lockdown period, we could only assess key HAI such as bacterial pneumonia, urinary tract infections and bloodstream infection. These are the same HAI reported in other publications regarding the Covid-19-lockdown [18, 19, 21].
4. Statistically, we compared a lockdown period including political ban of elective surgeries with normal periods. This study is the largest regarding Covid-19 lockdown and orthopedic surgery. The number of surgeries in the control periods are sufficient, but the lockdown period of six weeks was marked with a ban of elective surgeries. Hence, no single center with a ban of elective surgeries can catch up to an equivalent sample size in the lockdown and control periods. Such a formal analysis is only possible in multicenter evaluations or in settings without ban of elective surgeries during the first lockdown.
5. Finally, we did not monitor antibiotic consumption [19, 28–30] or the transmission of multi-resistant pathogens [19] because of the major biases induced by the lockdown and the presence of underpowered data in our single-center evaluation.

## Conclusion

In a large Swiss single-center cohort of 5791 adult orthopedic surgeries in a tertiary university hospital, the risks for deep SSI, wound healing disorders, HAI and other complications during the first total Covid-19 lockdown was not different to time periods pre- and post-lockdown. The public health measures did not influence our intra-hospital nosocomial epidemiology.

## Data Availability

The analyzed dataset from this investigation is available from the corresponding author on reasonable request.

## Abbreviations

ASA: American Society of Anesthesiology-score
BMI: Body mass index
CI: Confidence interval
CLABSI: Catheter-related bloodstream infection
HAI: Healthcare-associated infection
HCW: Healthcare worker
HH: Hand hygiene
HR: Hazard ratio
SARS-CoV-2: Covid-19
SD: Standard deviation
SSI: Surgical site infection
